# YODA Kinase Controls a Novel Immune Pathway of Tomato Conferring Enhanced Disease Resistance to the Bacterium *Pseudomonas syringae*

**DOI:** 10.3389/fpls.2020.584471

**Published:** 2020-10-14

**Authors:** Julio Téllez, Antonio Muñoz-Barrios, Sara Sopeña-Torres, Amanda F. Martín-Forero, Alfonso Ortega, Rosa Pérez, Yolanda Sanz, Marisé Borja, Alberto de Marcos, Michael Nicolas, Torben Jahrmann, Montaña Mena, Lucía Jordá, Antonio Molina

**Affiliations:** ^1^Centro de Biotecnología y Genómica de Plantas (CBGP, UPM-INIA), Universidad Politécnica de Madrid (UPM) – Instituto Nacional de Investigación y Tecnología Agraria y Alimentaria (INIA), Madrid, Spain; ^2^Departamento de Biotecnología-Biología Vegetal. Escuela Técnica Superior de Ingeniería Agronómica, Alimentaria y de Biosistemas, Universidad Politécnica de Madrid (UPM), Madrid, Spain; ^3^Facultad de Ciencias Ambientales y Bioquímica, Universidad de Castilla-La Mancha, Toledo, Spain; ^4^Plant Response Biotech, Centro de Empresas, Madrid, Spain; ^5^Semillas Fitó, Barcelona, Spain; ^6^Plant Molecular Genetics Department, Centro Nacional de Biotecnología/CSIC, Madrid, Spain

**Keywords:** Mitogen-activated protein kinases cascades, disease resistance, YDA, breeding, tomato, bacteria, plant immunity, stomata

## Abstract

Mitogen-activated protein kinases (MAPK) play pivotal roles in transducing developmental cues and environmental signals into cellular responses through pathways initiated by MAPK kinase kinases (MAP3K). AtYODA is a MAP3K of *Arabidopsis thaliana* that controls stomatal development and non-canonical immune responses. *Arabidopsis* plants overexpressing a constitutively active YODA protein (*AtCA-YDA*) show broad-spectrum disease resistance and constitutive expression of defensive genes. We tested YDA function in crops immunity by heterologously overexpressing *AtCA-YDA* in *Solanum lycopersicum*. We found that these tomato *AtCA-YDA* plants do not show developmental phenotypes and fitness alterations, except a reduction in stomatal index, as reported in *Arabidopsis AtCA-YDA* plants. Notably, *AtCA-YDA* tomato plants show enhanced resistance to the bacterial pathogen *Pseudomonas syringae* pv. *tomato* DC3000 and constitutive upregulation of defense-associated genes, corroborating the functionality of YDA in tomato immunity. This function was further supported by generating CRISPR/Cas9-edited tomato mutants impaired in the closest orthologs of *AtYDA* [*Solyc08g081210* (*SlYDA1*) and *Solyc03g025360* (*SlYDA2*)]. *Slyda1* and *Slyda2* mutants are highly susceptible to *P. syringae* pv. *tomato* DC3000 in comparison to wild-type plants but only *Slyda2* shows altered stomatal index. These results indicate that tomato orthologs have specialized functions and support that YDA also regulates immune responses in tomato and may be a trait for breeding disease resistance.

## Introduction

Plants rely on a two-tiered immune system to cope with the countless attempts of pathogens and pests to attack and colonize them. The first layer of this immune system consists of an extensive battery of membrane bound receptors, mainly receptor like kinases (RLKs) and receptor like proteins (RLPs), that are classified as pattern recognition receptors (PRRs). Successful recognition by these PRRs of highly conserved molecules from the pathogens (so-called, microbe-associated molecular patterns, MAMPs) activates MAMP-triggered immunity (MTI) that contributes to basal resistance by inhibiting pathogens colonization (Boutrot and Zipfel, 2017). Notably, PRRs can also perceive host (plant-self) derived molecules synthesized (e.g., peptides) or released upon pathogen infection or herbivory damage on plant tissues (e.g., plant cell wall derived glycans), that are referred as damage-associated molecular patterns (DAMPs), and activate complementary immune responses (DAMP-Triggered Immunity, DTI; Li et al., 2020). Pathogens have evolved mechanisms to inhibit immune responses by injecting effector proteins in plant cells to abolish defense signaling and resistance activation. To cope with this, plants have developed a second surveillance mechanism, named effector-triggered immunity (ETI), to sense these pathogen effectors through cytoplasmic PRRs encoded by the known resistance genes, which activate a more durable resistance response, that has been extensively used in crop protection breeding programs (Thordal-Christensen, 2020).

During MTI, DTI, and ETI activation, defense signaling is transmitted from the receptors to the nucleus, where a profound transcriptomic reprograming takes place, leading to the expression of genes that contributes to different resistance mechanisms [e.g., synthesis of antimicrobial metabolites and peptides/proteins, like pathogenesis related (PR) proteins, cell wall remodeling, and callose deposition; Albert et al., 2020]. Among the early downstream defense responses that are activated upon recognition of MAMP/DAMPs or effectors by PRRs are the production of reactive oxygen species (ROS), membrane ion fluxes, and activation of mitogen-activated protein kinases cascades (MAPKs or MPKs; Boutrot and Zipfel, 2017). MAPK cascades are central hubs in the plant immune signaling transduction since phosphorylation of proteins is one of the post-translational modifications, playing a major role in immune regulation and cell transcriptional reprograming (Bi and Zhou, 2017). Three levels of hierarchy operate in MAPK activation: MAPK kinase kinases (MAP3K/MEKKs) phosphorylate and activate MAPK kinases (MAPKK/MKKs) that later phosphorylate MAPKs. Activation of MAPKs will lead to phosphorylation of downstream targets, such as transcription factors or metabolic enzymes like those involved in synthesis of defensive phytohormones [e.g., salicylic acid (SA); Thulasi Devendrakumar et al., 2018].

In *Arabidopsis* and tomato, few PRR-MAPK modules involved in immunity have been described. For example, the FLAGELLIN-SENSITIVE 2 receptor (FLS2) that specifically recognizes the immunogenic MAMP peptide flg22, derived from the bacterial flagellin protein, activates a conserved cascade that involves the MAPKKK5, MKK4/MKK5, and MPK3/MPK6 (Liang and Zhou, 2018). Also, the signaling module composed by MEKK1, MKK1/MKK2, and MPK4 has been identified to be relevant in MTI (Ichimura et al., 2006; Suarez-Rodriguez et al., 2007; Gao et al., 2008; Kong et al., 2012). This cascade contributes to basal resistance and is guarded by the resistance (R) protein, SUMM2 (Zhang et al., 2012). Remarkably, this last mechanism highlights the relevance of MAPK cascades in immunity, as plants have developed a surveillance system to monitor that phosphorylation cascades are functional.

YODA (YDA) is a MAP3K from *Arabidopsis thaliana* that belongs to the MEKK family and together with MKK4/MKK5 and MPK3/MPK6 constitute a regulatory module controlling stomatal development (Bergmann et al., 2004). Perception of the extracellular peptides EPF1 and EPF2 by the receptor complex formed by the RLKs ERECTA (ER), ER-like family members ERL1 and ERL2, and brassinosteroid-associated kinase 1 (BAK1), and by the RLP Too Many Mouths (TMM), leads to a inhibition of stomatal development through the activation of YDA MAPK module (Lee et al., 2015; Meng et al., 2015). Accordingly, *Arabidopsis yda* mutants show an increase of leaf stomata and stomatal indexes (Bergmann et al., 2004; Sopeña-Torres et al., 2018). Remarkably, this ER/ERLs/TMM/BAK1-YDA signaling cascade has been demonstrated recently to operate also in *Arabidopsis* immunity, as mutants impaired in any of these molecular components are more susceptible to pathogens and are impaired in the activation of non-canonical immune pathways (Jordá et al., 2016; Sopeña-Torres et al., 2018). ER also controls in crops (e.g., tomato) other developmental processes, like internode length, rapid cycling, and compact plant size (Kwon et al., 2020), and some responses to stresses, such as thermotolerance (Shen et al., 2015).

N-terminal domain of some MAP3K has been shown to have a regulatory function on MAPKs activity. In fact, deletions of the N-terminal amino acids result in the constitutive activation of MAP3K (CA-MAP3K) based on the phenotypes observed in CA-MAP3K overexpressor lines. Tobacco and tomato plants expressing CA-MAP3Ks, such as tobacco MPK3α, show increased resistance to some pathogens, like the bacterium *Pseudomonas syringae*, but this phenotype was associated to the activation of programed cell death (PCD) that impacted negatively on plant fitness (Asai et al., 2002; del Pozo et al., 2004). Similarly, deletion of 184–322 amino acids of the N-terminal domain in *Arabidopsis* YDA (*AtCA-YDA*), leads to its constitutive activation and to a reduction of stomatal production in *Arabidopsis* plants (Lukowitz et al., 2004). Notably, *AtCA-YDA* plants are also highly resistant to a wide range of pathogens, including necrotrophic fungus, hemibiotrophic bacteria, and biotrophic oomycetes. *Arabidopsis AtCA-YDA* plants show constitutive expression of defensive genes that are not regulated by canonical immune pathways, like those mediated by defensive phytohormones, such as SA, jasmonic acid (JA), ethylene (ET), or MTI. Notably, *AtCA-YDA* plants do not show activation of PCD. These data led to suggest that YDA and their upstream PRRs (ER, ERL1, ERL2, TMM, and BAK1) regulate a novel non-canonical immune pathway conferring broad-spectrum disease resistance in *Arabidopsis* that could be of interest for breeding broad-spectrum disease resistance in crops (Molina et al., 2016; Sopeña-Torres et al., 2018).

To test whether YDA might play similar functions in crops immunity and to determine its biotechnological potential in breeding disease resistance, we overexpressed *AtCA-YDA* (*35S::AtCA-YDA*) in tomato (*Solanum lycopersicum*). Here, we show that constitutive expression of *AtCA-YDA* in tomato confers enhanced resistance against the bacterium *P. syringae* pv. *tomato* (*Pto*), supporting that YDA modulates immune responses in other plant species. The stomatal index was also found to be reduced in tomato *35S::AtCA-YDA* lines further corroborating the functionality of YDA in stomatal regulation in crops. We also proved YDA function in tomato by generating CRISPR/Cas mutant lines in *SlYDA1* and *SlYDA2*, the two closest orthologs of *AtYDA*, which were found to be highly susceptible to *Pto*, further demonstrating the relevant role of *YDA* in crops immunity.

## Materials and Methods

### Plant Growth Conditions

The Moneymaker (MM) cultivar of tomato (*S. lycopersicum*) was used as wild type in all the assays performed. Seeds were sown individually in pots with a mixture of peat moss:vermiculite (3:1). Plants grew at 24°C in the greenhouse with a light regime of 12 h. For *in vitro* growth assays, seedlings were surface sterilized by incubating them in bleach (100%) for 4 min, washed with sterilized water four times, and then plated onto MS media (Murashige and Skoog basal salt medium; Duchefa Biochemie).

### Stable Transformation of *Solanum lycopersicum*

Moneymaker cultivar was transformed with *Agrobacterium tumefaciens* carrying the binary vector pGWB2 for *AtYDA* (*CaMV35S::AtYDA*; Nakagawa et al., 2007) and pB2GW7 for *AtCA-YDA* (*CaMV35S::AtCA-YDA*; Karimi et al., 2002) at the transformation service of University of California at Davis (United States) using its standard protocol.^1^ *AtYDA* tomato transgenic plants were selected on kanamycin/hygromycin plates (50 and 10 mg/l, respectively; Duchefa Biochemie), while *AtCA-YDA* plants were selected based on their resistance to the glufosinate herbicide. Homozygous lines were selected by PCR and the levels of transgene expression were also measured by qRT-PCR using the oligonucleotides depicted on Supplementary Table S9. Two independent homozygous lines transformed with *AtCA-YDA* were selected (#3 and #7) and two for *AtYDA* (#6 and #5). *AtYDA* line #5 does not show expression of the transgene and was used as control line. All the assays were performed with T3 plants.

### Generation of CRISPR/Cas9 Lines of *Solanum lycopersicum*

Tandem gRNAs targeting two closely located sites were designed for both *SlYDA1* and *SlYDA2* under control of different U6 promoters. gRNAs were designed using the “Breaking Cas” online tool (Oliveros et al., 2016).^2^ *SlYDA1* gRNAs (5'-CGAGCTGTCAGGAGCACTGT-3' and 5'-TGAACCCTGGTTTCTGGCTA-3') had an aggregated score of 98.9 (100 maximum score) and are targeting the third exon. *SlYDA2* gRNAs (5'-CCTTCTTTTAGGAGATACGG-3' and 5'-CTGCAAAAGGCTCCAGGAGG-3') had an aggregated score of 97.5 and 99.5, respectively, and are targeting the second exon. All the potential off-targets had an individual score ≤0.5 with at least four mismatches within the 20-nt gRNA sequence selected. The tandem gRNAs for each gene were cloned together with the UBQ10/CAS9 sequence through triple LR cloning (Gateway system, Life Invitrogen) into the pB7m34GW,0 vector, which allow selection of the transgenic plants through BASTA/PPT. Both constructs have been used to transform MM cultivar using *A. tumefaciens* at the transformation service of the Universidad Politécnica de Valencia (UPV) following their standard transformation protocol. Transformants were selected on BASTA resistance, transplanted, and selfed for T2 seed production. T2 seeds were sown and selected in plantlet state by HRM PCR (High Resolution Melting; Roche LightCycler 480) for the presence of CRISPR induced mutations, based on the sequences of the gRNAs. Additionally, PCR analysis was performed to test the absence of the transgene. Selected plants were transplanted and selfed for T3 seed production. T3 plants were analyzed by Sanger sequencing (Macrogen sequencing Service) to confirm the homozygosity of the mutation and to characterize the modification. Based on the CRISPR/Cas9 induced mutations, CRISPR mutants #36 and #37 (two independent *Slyda1* mutant lines of *SlYDA1*) and #23, #52, and #313 (three independent *Slyda2* mutant lines of *SlYDA2*) were selected for further analysis.

### Morphometric and Stomatal Abundance Parameters

As the fruits grew and ripened, they were picked and immediately their fresh weight was determined. Pictures of the tomato plants were taken at 18, 25, and 35 days after germination. Stomatal and pavement cell densities and stomatal indexes were scored in the terminal leaflet of the third leaf of 25-day-old plants. Leaflets were hand-excised, fixed, and clarified as indicated in Morales-Navarro et al. (2018) with minor modifications. Plant material was fixed in ethanol:acetic acid 9:1 (v/v) for 48 h, rehydrated through ethanol series, and cleared in a chloral hydrate:glycerol:water solution (8:1:2, w/v/v) for 48 h. The abaxial epidermis was inspected with differential interference contrast (DIC) under a Nikon Eclipse 90i upright microscope and a DXM1200C camera for image acquisition. Micrographs were analyzed with the free software Fiji Image J (Schindelin et al., 2012). Epidermal cell counts of each individual were an average from two areas of 0.4 mm^2^. Stomatal and pavement cell density were calculated as number of stomata or pavement cells per area unit (cell number mm^−2^), respectively, and the stomatal index as percentage of epidermal cells that were stomata (number of stomata/total number of epidermal cells × 100).

### Pathogenicity Assays

*Pseudomonas syringae* pv. *tomato* DC3000 was grown for 36 h in King Agar B (KB) plates under dark conditions. Cells were scraped from the plates and suspended in 10 mM MgCl_2_ at a final concentration of 10^8^ colony forming units (CFU)·ml^−1^, and 0.002% (vol/vol) Silwet L-77 was added prior inoculation. Three-week-old plants of wild-type genotype and of each transgenic (#3 and #7 of *AtCA-YDA*, #5 and #6 of *AtYDA*) or CRISPR/Cas9 mutant lines (#36 and #37 of *Slyda1* and #23, #52, and #313 of *Slyda2*) were spray-inoculated with the bacterial suspension. Bacterial growth was determined at 3 h post inoculation (time 0) and 5 days post infection by averaging the CFU·cm^−2^ isolated from four infected plants. Leaf disks were homogenized in 10 mM MgCl_2_, and serial dilutions were plated on KB solid medium containing rifampicin (25 μg/ml; Duchefa Biochemie). These assays were repeated in triplicate.

### RNA Extraction for Gene Expression Analyses

Total RNA was isolated from tomato leaves of at least three plants at the indicated days after sowing using an extraction buffer (0.2 M Tris pH 9; 0.4 M LiCl; 25 mM EDTA, and 1% SDS), followed by two phenol extraction steps and an 8 M LiCl precipitation. Two milligram of total RNA resuspended in milli-Q water was treated with DNaseI following the manufacturer’s protocol (TURBO DNAfree, Ambion). First-strand cDNA synthesis was performed with oligo-dT using Transcriptor First Strand cDNA Synthesis (Roche) following manufacturer instructions. SYBRGreen master mix (Roche) was used for qRT-PCR reactions that were performed in the LightCycler® 480 Instrument II (Roche) as reported previously (Jordá et al., 2016). *UBC* (*Solyc03g123660*) was used as tomato housekeeping gene. Primers used for expression analysis are listed in Supplementary Table S9. All the gene expression assays were performed at least three times.

### RNA-seq Analyses

The terminal leaflet of the third leaf of 25-day-old plants was used to extract RNA. Total RNA was isolated as described in the previous section, from four independent individuals of MM, *AtCA-YDA#3*, *AtCA-YDA#7*, and *AtYDA#6* lines, from two independent experiments, and subjected to RNA sequencing (*n* = 4 for *AtCA-YDA* lines and *n* = 2 for *AtYDA* lines). RNA was further purified using the RNeasy Mini Kit following manufacturer instructions (Qiagen). RNA samples were sequenced in the sequencing Core Facilities of the Center for Regulatory Genomics, CRG (Barcelona, Spain). Prior to sequencing quality control of RNA samples was performed with a Bioanalizer. Samples were sequenced with the Illumina HiSeq3000 (high output mode) with x50 tomato genome coverage using 1 × 50 bp reads. Raw data were aligned to the newly annotated *S. lycopersicum* genome with HISAT2 v2.1.0 (Kim et al., 2015). Then it was sorted with Samtools (Li et al., 2009) and differential expression was obtained with StringTie (Frazee et al., 2015; Pertea et al., 2015). Functional categories of the missregulated genes were obtained using Blast2Go pipeline (Götz et al., 2008) of the whole published annotation (data base from NCBI December 06, 2017). Gene ontology categories were done using the BINGO program (Maere et al., 2005).

### Phylogenetic Analyses/Ortholog Search

MEGA5 program (Tamura et al., 2011) was used to perform the evolutionary analysis. The full length protein sequence of *Ar. thaliana At*YODA (At1g63700), the putative orthologs from *S. lycopersicum* (*Sl*YDA1, Solyc08g081210; *Sl*YDA2, Solyc03g025360; and *Sl*YDA3, Solyc06g03680) and some Solanum MAP3K (Solyc12g088940, Solyc09g047920, Solyc04g079400, Solyc02g090430, Solyc02g065110, Solyc04g064590, and Solyc03g117640) were aligned using MUSCLE and inferred using the neighbor-joining method. The protein sequence of Solyc06g005910 (tubulin β-chain) was used to root the tree. Identity and similarity calculations between the protein sequences were determined using Sequence Identity And Similarity (SIAS) tool and BLOSUM62 matrix.^3^

### Statistical Methods

Normality of distributions was analyzed using Shapiro-Wilk normality test. Student’s *t*-test or the Mann-Whitney test analyses were performed with STATGRAPHICS CENTURION XVI (Statpoint Technologies, Warrenton, VA, USA).

### Accession Numbers

RNA-seq read data have been submitted to the NCBI Sequence Read Archive (SRA) under accession PRJNA635132.

## Results

### Expression of *AtCA-YDA* in Tomato Does Not Affect Plant Fitness

**Figure 1:**
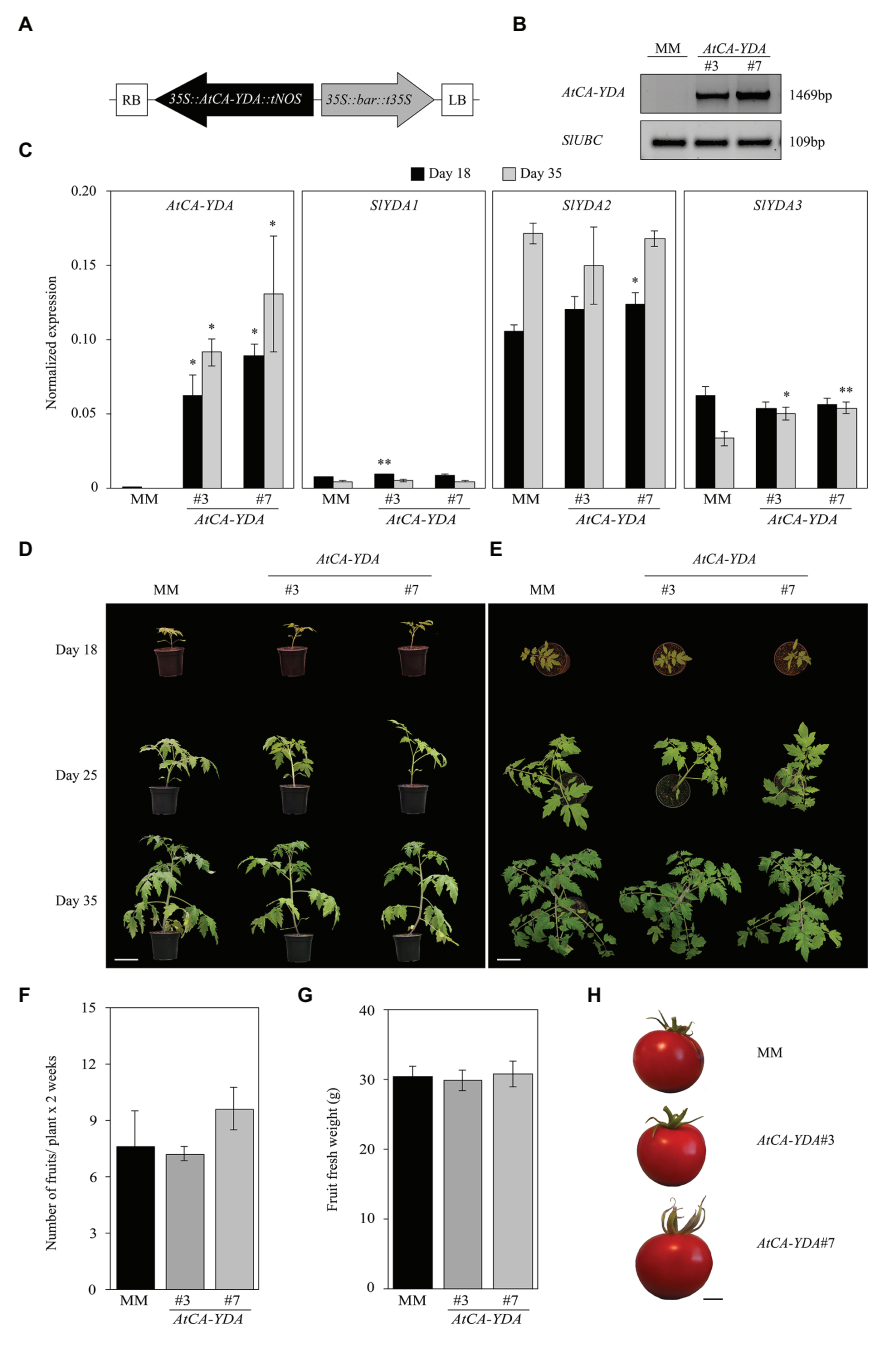
Expression of *AtCA-YDA* in tomato does not have deleterious effect on plant development. **(A)** Scheme of construction used for transformation of tomato plants with *AtCA-YDA* in pB2GW7 vector, driven by the *CaMV 35S promoter* (*35S*) and nopaline synthase terminator (*tNOS*). RB, right border sequence of T-DNA; LB, left border sequence of T-DNA; *bar* cassette to express the DL-phosphinothricin resistance gene driven by *35S* and terminator *t35S*. **(B)** PCR detection of the transgene on Moneymaker (MM) wild-type plants and transgenic lines *AtCA-YDA #3* and *#7*. *SlUBC* gene was PCR amplified and used as equal DNA loading control. **(C)** Expression of *AtCA-YDA*, *SlYDA1* (*Solyc08g081210*), *SlYDA2* (*Solyc03g025360*), and *SlYDA3* (*Solyc06g036080*) in *S. lycopersicum* MM and transgenic *AtCA-YDA* plants (lines #3 and #7) at 18 and 35 days after sowing. Shown data are average expression of three independent experiments (*n* = 3) ± SE. Asterisks indicate statistical significance with respect to MM plants (Student’s *t*-test; ^*^p < 0.05 and ^**^p < 0.01). **(D,E)** Morphology of the indicated genotypes at 18, 25, and 35 days after sowing. Bar = 10 cm. **(F)** Number of fruits collected from the indicated lines (*n* = 12). **(G)** Fresh weight (grams/fruit) of the tomato fruits produced by *AtCA-YDA #3* (*n* = 36) and *#7* (*n* = 48) and MM plants (*n* = 58). These results are representative of at least three different experiments performed that gave similar results. **(H)** Representative phenotypes of fruits of MM and *AtCA-YDA #3* and *#7* plants. Bar = 1 cm.

Since overexpression of *AtCA-YDA* in *Arabidopsis* confers broad-spectrum disease resistance to pathogens and activates specific immune responses (Sopeña-Torres et al., 2018), we explored if CA-YDA protein could be also a positive regulator of immune responses in other plant species, like tomato (*S. lycopersicum*). We first heterologously expressed *AtCA-YDA* in tomato (MM) by generating plants constitutively overexpressing *AtCA-YDA* that were obtained by cloning *AtCA-YDA* coding sequence (harboring a deletion of the amino acids 184–322 at the N-terminal region) into the binary vector pB2GW7 (Karimi et al., 2002) under the control of the 35S cauliflower mosaic virus promoter (*35SCaMV; 35S::AtCA-YDA* lines; Figure 1A). Among the plant transformants generated, we selected for further characterization two independent *AtCA-YDA* tomato lines (*AtCA-YDA* #3 and *AtCA-YDA* #7) harboring a single insertion event based on segregation analyses. We determined the presence of the *AtCA-YDA* transgene on the transformant lines by PCR (Figure 1B), and we proved by qRT-PCR that *AtCA-YDA* was expressed in leaves of the transgenic lines at different developmental stages (Figure 1C). Since expression levels of MAP3K are fine-tuned, we assessed whether overexpression of *AtCA-YDA* could impact on the expression of the three *S. lycopersicum YDA* ortholog genes (*Solyc03g025360*, *Solyc06g036080*, and *Solyc08g081210*; Supplementary Figure S1). Phylogenetic analysis showed that *Solyc08g081210* (*SlYDA1*), *Solyc03g025360* (*SlYDA2*), and *Solyc06g036080* (*SlYDA3*) are the closest *AtYDA* orthologs based on the alignment of full length proteins (Supplementary Figure S1), and their similarities to *At*YDA ranged from 67.5% of *Sl*YDA1 to 65.23% of *Sl*YDA3 (Supplementary Figure S2). *Solyc03g025360* and *Solyc06g036080* (*SlYDA2* and *SlYDA3*) seem to result from a gene duplication (Supplementary Figures S1, S2A). The N-terminal sequence of *Sl*YDA2 showed higher similarity with the N-terminal of *At*YDA than that of *Sl*YDA1 that has some amino acid deletions in comparison to *At*YDA sequence (Supplementary Figure S2A).

Expression levels of these three genes, determined by qRT-PCR, were found to be very low in tomato leaves of wild-type MM plants. Overexpression of *AtCA-YDA* in the transgenic lines has minor effect on the transcription levels of *SlYDA1* or *SlYDA2*, at any of the developmental stages analyzed, whereas *SlYDA3* gene exhibited slight higher expression levels in these lines at 35 days post seed germination than in wild-type plants (Figure 1C).

We next assessed whether *AtCA-YDA* expression had any impact on tomato development. For that purpose, we monitored the growth of these plants and MM throughout their development and evaluated the production of fruits. We found that neither of the two tomato *AtCA-YDA* lines showed a penalty on plant or fruit development nor reduction in fruit yield per plant compared to wild-type (MM) plants (Figures 1D–H). We also tested the impact of *AtCA-YDA* overexpression on *in vitro* seed germination since YDA has been described to regulate *Arabidopsis* embryo development (Bergmann et al., 2004). We observed that seeds of the two independent *AtCA-YDA* lines showed a slight delay in reaching 100% germination in comparison to wild-type (MM) seeds (Supplementary Figure S3A), but they did not show any alteration in root or leaf development (Supplementary Figure S3B).

We also generated for comparison *35S::AtYDA* tomato transgenic lines expressing the full-length protein by cloning the *AtYDA* full sequence into pGWB2 vector under the control of *35SCaMV* (*35S::AtYDA* lines; Nakagawa et al., 2007; Supplementary Figure S4A). Several transformed lines were selected for further characterization, but after extensive analyses of *AtYDA* expression, we only identified one independent *35S::AtYDA* line (*AtYDA* #6) with detectable expression levels of the transgene (Supplementary Figure S4C). Also, we selected as negative control for the analyses, an stable transformant line (*AtYDA* #5) that did not express the transgene (Supplementary Figures S4B,C). The expression of tomato *SlYDA1-SlYDA3* genes in the *35S::AtYDA* lines was not affected, with the exception of *SlYDA3*, whose expression was, like in the *35S::AtCA-YDA* tested (Figure 1C), slightly higher in the transgenic line than in wild-type plants (Supplementary Figure S4C). Besides, no developmental phenotypic alterations and fruit yield penalties were found on the *AtYDA* #6 line expressing the transgene (Supplementary Figures S4D–G).

### *AtCA-YDA* Expression Modulates Stomatal Development in *Solanum lycopersicum*

**Figure 2:**
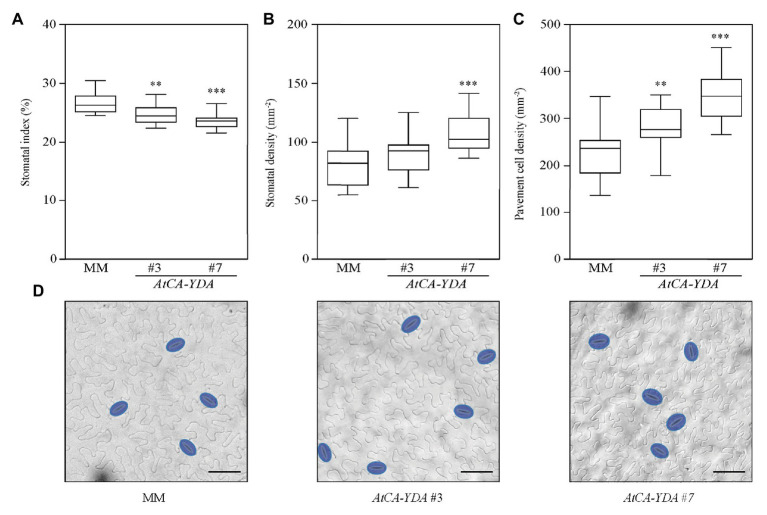
Tomato plants overexpressing *AtCA-YDA* show reduced stomatal index and higher epidermal cell density. **(A–C)** Stomatal index, stomatal density, and pavement cell density were analyzed in the abaxial epidermis of the mature third leaf from wild-type (MM) and transgenic *AtCA-YDA* plants (lines #3 and #7). The box plot diagram shows the median of the distribution as a center line, and the 25th and 75th percentiles are indicated by box limits. Whiskers show the minimum and maximum values. Data were obtained from 11 to 16 plants of each genotype. Asterisks indicate statistical significance with respect to MM plants (Student’s *t*-test; ^**^p ≤ 0.01 and ^***^p ≤ 0.001). **(D)** Representative differential interference contrast (DIC) images of the epidermis scored in panels **(A–C)** leaves of plants from MM and two independent *AtCA-YDA* lines (#3 and #7), as indicated in the panels. Stomata are false-colored in blue. Bar = 50 μm. This experiment was performed twice and gave similar results. See also Supplementary Table S1.

*AtYDA* regulates stomatal development and pattern in *Arabidopsis*. Guard cell formation is completely abolished in *Arabidopsis AtCA-YDA* homozygous plants (Lukowitz et al., 2004). To unveil, whether *AtCA-YDA* overexpression could lead to similar phenotypes on tomato, we analyzed stomatal abundance in the lines transformed with *AtCA-YDA* (Figure 2; Supplementary Table S1). The stomatal index (percentage of epidermal cells that are stomata) was reduced in the *AtCA-YDA* lines compared to the MM control plants (Figure 2A), indicating that expression of a constitutively active YDA protein was able to control the cell fate of tomato epidermal cells, repressing stomatal production as reported in *Arabidopsis* (Lukowitz et al., 2004). Significantly, the *AtCA-YDA* lines showed increased stomatal and pavement cell density compared to MM control plants (Figures 2B,C), indicating additional effects of *AtCA-YDA* on epidermal cell size and probably in the specification of epidermal cell fate. These results confirm that *AtCA-YDA* is functional in *S. lycopersicum* as stomatal development is affected by its constitutive overexpression.

### *AtCA-YDA* Tomato Plants Are More Resistant Than Wild-Type Plants to the Bacterium *Pseudomonas syringae* pv. *tomato*

**Figure 3:**
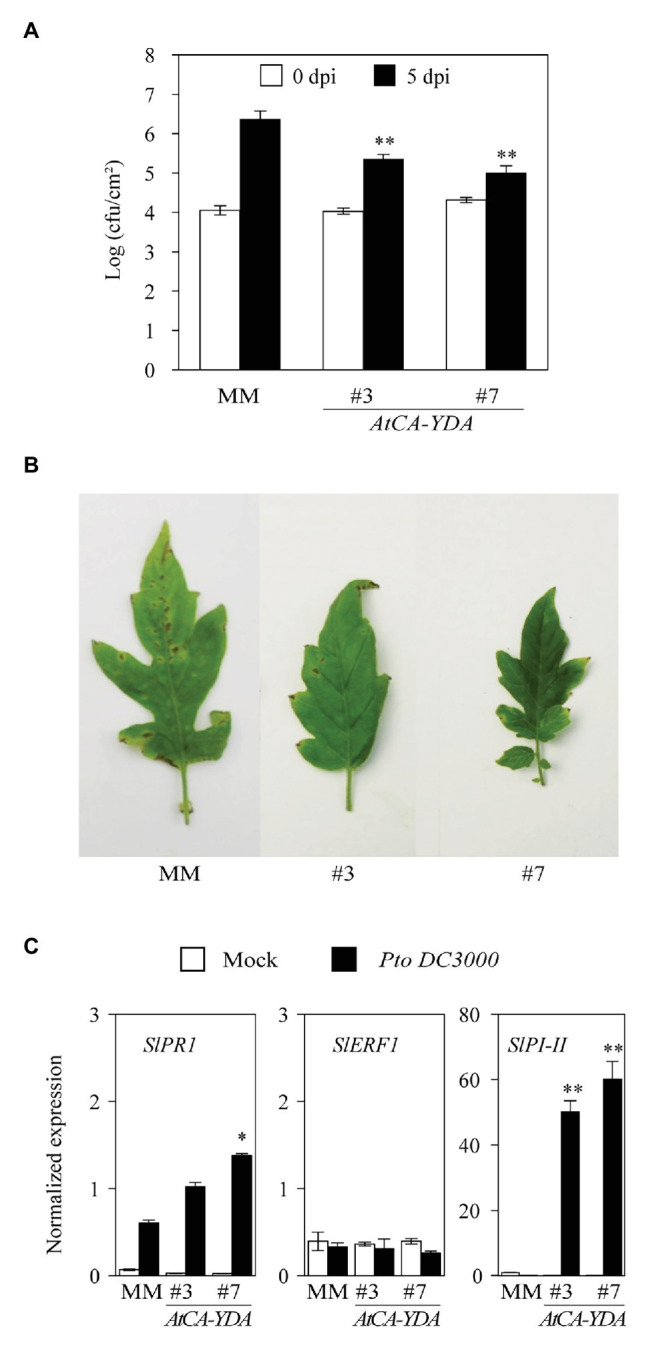
Tomato plants expressing *AtCA-YDA* show enhanced resistant to the pathogenic bacteria *Pseudomonas syringae* pv. *tomato* DC3000. **(A)** Quantification of bacterial growth at 0‐ and 5-day post-inoculation (dpi) with 10^8^ cfu/ml of the bacterium. Values represent the mean (*n* = 12 ± SE) of three independent experiments. Asterisks indicate statistical significance with respect MM inoculated plants (Student’s *t*-test; ^**^p < 0.01). **(B)** Macroscopic symptoms of bacteria inoculated leaves of the indicated genetic genotypes at 8 dpi. **(C)** Expression of *SlPR1*, *SlERF1*, and *SlPI-II* in response to *Pto* DC3000 inoculation (10^8^ cfu/ml) or mock treatment (10 mM MgCl_2_). Leaf samples were collected at 24 hours post inoculation (hpi) and gene expression determined. *SlUBC* (*Solyc03g123660*) was used as internal reference gene. Data are the means (*n* = 4 ± standard errors) of the normalized gene expression. Statistical significance compared with MM plants was determined by Student’s *t*-tests: plants (^*^p < 0.05 and ^**^p < 0.01). This experiment has been performed at least three times with similar results.

We next tested whether constitutive activation of *AtYDA* on tomato plants can enhance resistance, as it has been described in *AtCA-YDA Arabidopsis* plants (Sopeña-Torres et al., 2018). With that purpose, we spray-inoculated 3-week-old plants of two *AtCA-YDA* tomato lines and wild-type (MM) genotype with a suspension of the virulent bacteria *P. syringae* pv. *tomato* DC3000 (*Pto*), and bacterial growth was monitored at 3 h post inoculation (0 days post-inoculation, dpi), for determining equal bacterial inoculation in the tested genotypes, and at 5 dpi to characterize bacterial growth and infection. Remarkably, bacterial colony forming units (cfu/cm^2^) in the two *AtCA-YDA* tomato lines were significantly lower than in the wild-type plants (Figure 3A). Decrease in the bacterial growth was also associated with a reduction of disease symptoms caused by bacterial infection, which were lower in tomato *AtCA-YDA* lines than in MM (Figure 3B). This enhanced resistance phenotype of *AtCA-YDA* tomato lines was not due to a constitutive activation of canonical defense signaling pathways required for resistance to *Pto* (Betsuyaku et al., 2018; Liu et al., 2019), like those mediated by SA or ethylene (ET) or jasmonic acid (JA), since expression of tomato marker genes of these pathways (e.g., *SlPR1*, *SlERF1*, and *SlPI-II*, respectively) was not constitutively upregulated in mock-treated plants, as determined by qRT-PCR (Figure 3C). However, expression levels of *SlPR1* and *SlPI-II* were higher at 24 hpi in *AtCA-YDA* lines than in inoculated wild-type plants (Figure 3C). Remarkably, *35S::AtYDA* line #6 overexpressing *AtYDA* was also found to be slightly more resistant to *Pto* than wild-type plants or the *35S::AtYDA #5* line (not expressing the transgene) used as negative control (Supplementary Figure S5). Together, our results indicate that heterologous expression of *AtCA-YDA* has a significant impact on tomato immune responses and that YDA-mediated immune pathway is also conserved in this crop, as previously reported in *Arabidopsis* (Sopeña-Torres et al., 2018).

### *AtCA-YDA* Expression in Tomato Induces the Transcriptional Activation of Defense Genes

**Figure 4:**
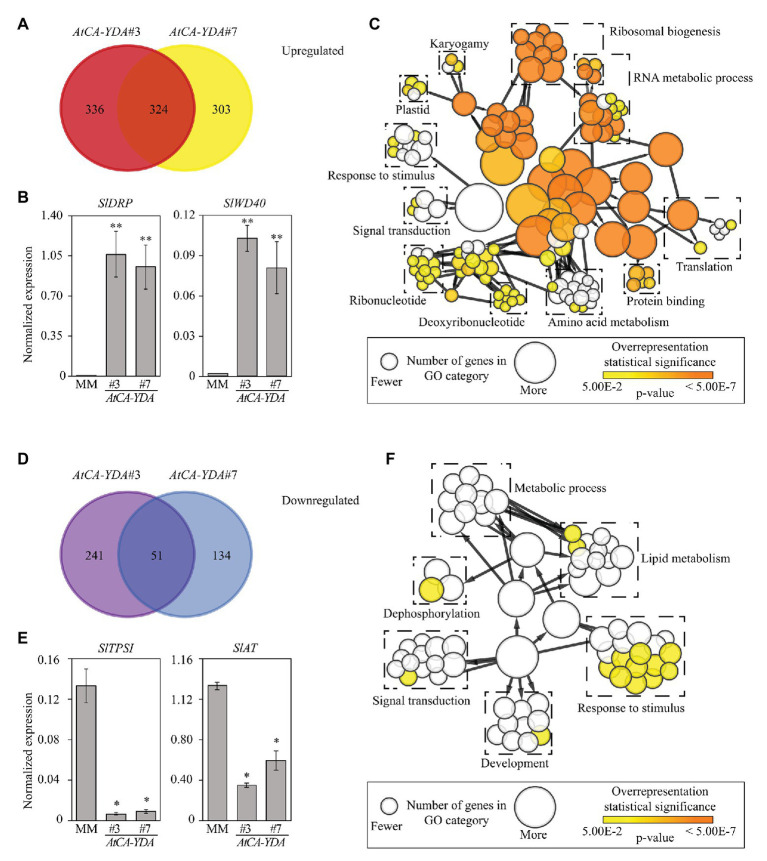
Expression of *AtCA-YDA* in tomato triggers the transcriptional regulation of defense genes. **(A)** Venn diagram showing overlapping of genes upregulated in *AtCA-YDA* lines (#3 and #7) compared to MM. Differential expression was defined as at least 2-fold change when compared with the non-transformed controls (MM). A minimal gene coverage value of 20 for each gene in RNAseq data was considered for selecting DEGs. **(B)** qRT-PCR analyses to validate the expression of upregulated genes in RNAseq data of *AtCA-YDA* plants. Expression levels of a disease resistance protein encoding gene (*Solyc02g077940*) and a transducin (*Solyc05g013880*) were analyzed and relativized to the *SlUBC* gene expression. Values are means (*n* = 6 ± SE) of the normalized gene expression. Statistical significance compared with MM plants was determined by Student’s *t*-tests (^*^p < 0.05 and ^**^p < 0.01). **(C)** Functional category classification of the 324 DEGs upregulated in *AtCA-YDA* tomato lines. BINGO pipeline was used to assign the categories. Each significantly enriched GO term is represented with a circle, and the contribution of which is related to its diameter as indicated at the bottom inset. Significant enrichments according to the value of *p* are depicted in orange using the color code shown at the bottom right inset. Supplementary Table S2 depicts the genes included in this study. **(D)** Venn diagram of the DEGs downregulated in *AtCA-YDA* lines (#3 and #7) vs. wild-type (MM) plants. **(E)** qRT-PCR analyses to validate the expression of downregulated genes in RNAseq data of *AtCA-YDA* plants: phosphate starvation-induced gene (*SlTPSI1*, *Solyc03g098010*) and acetyl transferase encoding gene (*SlAT*, *Solyc02g079490*). Expression levels are normalized to the *SlUBC* (*Solyc03g123660*) gene. Values are means (*n* = 4 ± SE). Asterisks indicate mean values significantly different from wild-type plants (MM; Student’s *t*-test; ^*^p < 0.05 and ^**^p < 0.01). All expression analyses were performed at least three times with similar results. **(F)** Functional category classification of the 51 DEGs downregulated in the *AtCA-YDA* tomato lines using BINGO pipeline. Diameter of the circles indicates number of genes on each GO cellular component category enrichment and orange color indicates that the overrepresentation is statistically significant (*p* < 0.05). Supplementary Table S3 shows the genes downregulated in *AtCA-YDA* lines.

To determine the basis of YDA-mediated resistance in tomato, we analyzed the transcriptomic reprograming that occurred in *AtCA-YDA* tomato transgenic lines. We performed a global RNA sequencing analysis (RNA-seq) on 4-week-old leaves from the two *AtCA-YDA* lines selected (#3 and #7) and wild-type plants. We identified a set of commonly differentially expressed genes (DEGs, *n* = 375, *p* ≤ 0.05, fold change ≥ 2) in the two *AtCA-YDA* lines tested in comparison to wild-type plants. Specifically, 324 DEGs were upregulated, while 51 were downregulated in both *AtCA-YDA* lines (Figures 4A,D; Supplementary Tables S2, S3). Functional classification of the DEGs identified some similarities between the functional categories of the genes upregulated both in tomato and *Arabidopsis CA-YDA* plants (Sopeña-Torres et al., 2018), like those related to translation, or RNA and ribosomal biogenesis, and response to stimuli (Figure 4C). Interestingly, other important category that is overrepresented in *AtCA-YDA* lines included defense-related genes, such as those encoding subtilisins, antimicrobial peptides, or resistance proteins (Figure 4C). qRT-PCR assays performed in independent experiments validated the differential expression of the upregulated genes tested, that encode proteins related with immunity, such as TIR-NBS-LRR disease resistance protein (*SlDRP*, *Solyc02g077940*) and Transducin (*SlWD40*, *Solyc05g013880*; Figure 4B). We also found 51 DEGs downregulated in both *AtCA-YDA* transgenic lines (Figure 4D), and two of them were validated by qRT-PCR (*SlTPSI*, *Solyc03g098010* and *SlAT*, *Solyc02g079490*; Figure 4E). The classification into functional categories of the downregulated DEGs in *AtCA-YDA* lines identified genes involved in response to stimuli, signaling, and lipid metabolism (Figure 4F).

We took advantage of the generation of the *35S::AtYDA #6* line to determine if transcriptional reprograming regulated by AtCA-YDA and AtYDA proteins were similar, since this comparison has not been reported so far in *Arabidopsis*. Global RNA sequencing analysis (RNA-seq) was performed with the *AtYDA #6* line. Of 934 genes found to be differentially expressed (777 up-expressed and 157 down-expressed) in this line (Supplementary Tables S4, S5) only 77 genes (43 upregulated and 34 downregulated) showed similar pattern of expression in *AtCA-YDA* lines, indicating that the transcriptional activation regulated by these two protein versions might be quite different (Supplementary Tables S6, S7). A core of 43 genes were found to be upregulated in *AtCA-YDA* and *AtYDA* lines, which were mainly grouped into gene ontology terms related with translation and defense, but also included genes with unknown function (Supplementary Figures S6A,B; Supplementary Table S5). Among the 34 genes downregulated in *AtCA-YDA* and *AtYDA* lines, we could identify a set of genes involved in stress responses, like phosphate starvation and defense response, including a gene of *MLO* family. This family has been demonstrated to have a negative function in the modulation of crops resistance to powdery mildew, that is enhanced in crops harboring *mlo* mutations (Kusch and Panstruga, 2017; Supplementary Figures S6C,D; Supplementary Table S7).

### Disruption of the Two Closest Orthologs of *AtYDA* Genes in Tomato (*SlYDA1* and *SlYDA2*) Leads to Enhanced Susceptibility to *Pseudomonas syringae* pv. *tomato* DC3000

**Figure 5:**
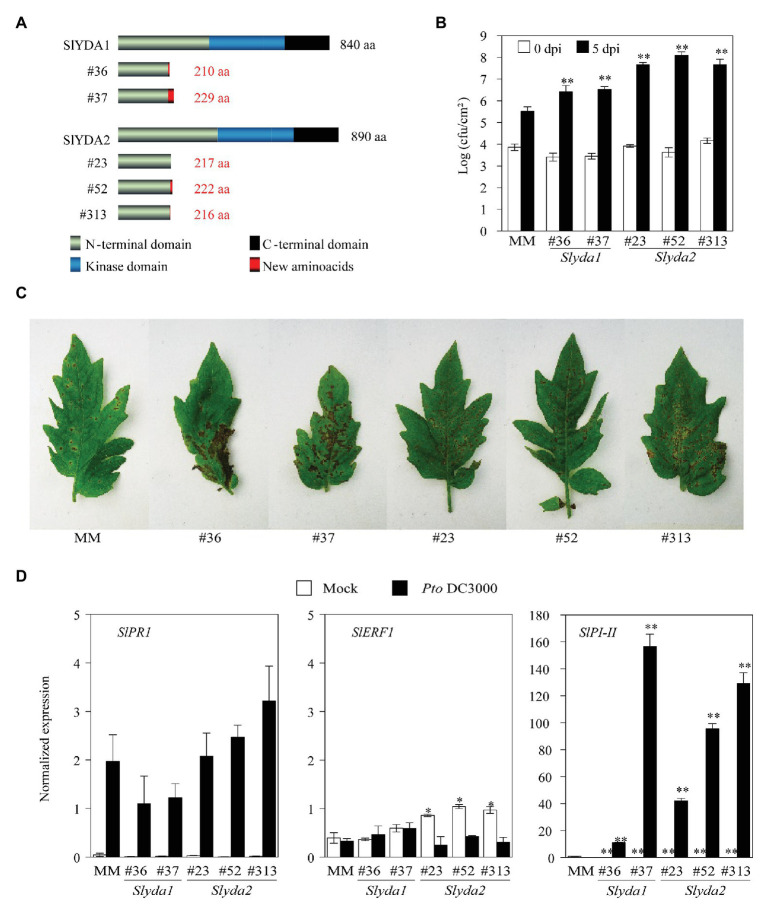
Mutations of *SlYDA* orthologs lead to enhanced susceptibility to *Pseudomonas syringae*. **(A)** Diagram of mutations generated in *SlYDA1* and *SlYDA2* genes by CRISPR/Cas9. **(B)** Growth of spray-inoculated *P. syringae* pv. *tomato* (*Pto*) DC3000 (10^8^ CFU ml^−1^) in 21-day-old tomato plants of the indicated genotypes. Bacterial numbers were determined at 0 and 5 dpi. Values represent the mean (*n* = 12) ± SE of three independent experiments. Asterisks indicate mean values significantly different from MM control plants (Student’s *t*-test; ^*^p < 0.05 and ^**^p < 0.01). **(C)** Macroscopic symptoms of *Pto* DC3000-inoculated leaves at 8 dpi. Experiments were performed three times with similar results. **(D)** Defense gene expression analysis in MM, *Slyda1*, and *Slyda2* plants mock‐ or bacterial-inoculated. Leaf samples were collected at 24 hpi and expression of genes was analyzed by qRT-PCR. Expression levels are relative to *SlUBC* gene. Values represent means (*n* = 6) ± SE from three independent biological replicates. Asterisks indicate mean values significantly different from expression in MM plants (Student’s *t*-test; ^*^p < 0.05 and ^**^p < 0.01).

To further confirm the relevance of YDA-mediated pathway in tomato disease resistance responses, we next assessed the role of the two closest *AtYDA* orthologs in tomato, *SlYDA1* and *SlYDA2* (Supplementary Figures S1, S2). For that purpose, we generated null mutants using CRISPR/Cas9 genome editing-technology in the Moneymaker tomato variety. Two independent mutant lines were obtained for *SlYDA1* (*Slyda1* Lines #36 and #37), while three CRISPR alleles were identified for *SlYDA2* (*Slyda2* Lines #23, #52, and #313). DNA sequence analysis allowed to confirm the mutations in these lines that lead to a shift in the open reading frame that resulted in premature stop codons and a truncated YDA protein in all the CRISPR/Cas9 lines generated (Figure 5A). Further analysis of *Slyda* lines showed that all mutations were stably transmitted to the offspring. Notably, mutations in *SlYDA1* led to plants with reduced height and biomass at early stages of their developmental cycle in comparison to wild-type plants, although *Slyda1* mutants were able to complete their cycle and set viable fruits. In contrast, mutations in *SlYDA2* had no detrimental impact on tomato development and fitness (Supplementary Figure S7).

We next evaluated the defense response of these *Slyda1* and *Slyda2* mutant lines to the bacterial pathogen *Pto* DC3000. Notably, mutations in both tomato *YDA* genes resulted in plants highly susceptible to this bacterium (Figure 5B) since the number of cfu/cm^2^, as well as leaf disease symptoms, were significantly enhanced in these mutants in comparison to wild-type plants (Figures 5B,C). This enhanced susceptibility was not due to an impairment in the induction of the expression of SA, ET, or JA-mediated defense genes, since the expression of the JA marker gene *SlPI-II* was enhanced in the *Slyda1/Slyda2* lines upon infection, probably due to a faster bacterial colonization of these plants in comparison to wild-type plants, whereas the expression of *SlPR1* and *SlERF1* upon infection did not differ from that of wild-type plants (Figure 5D). Moreover, the expression of these defensive marker genes in non-inoculated mutants did not differ from that of wild-type plants with the exception of *SlERF1* that showed a slightly higher expression in *Slyda2* lines (Figure 5D).

**Figure 6:**
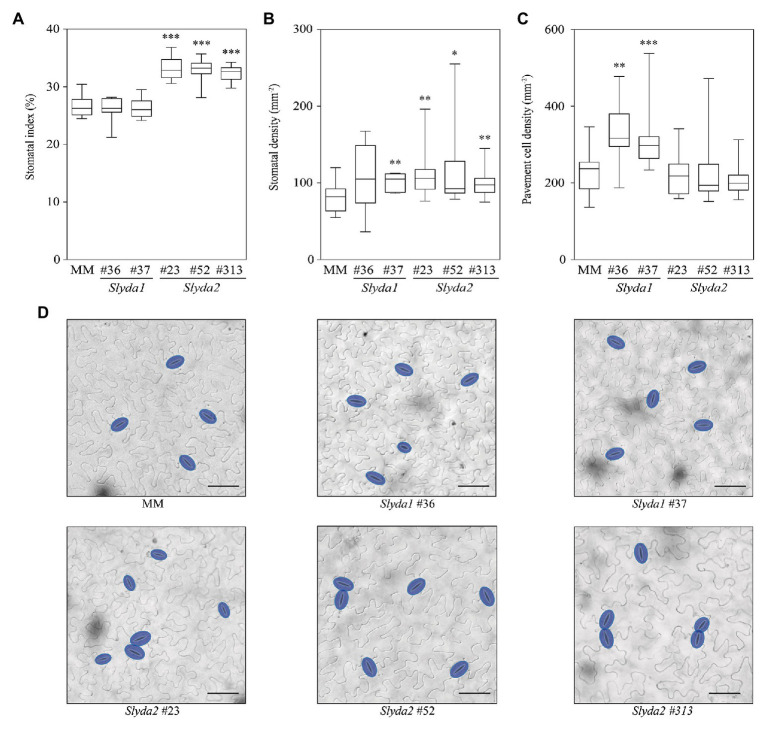
*SlYDA2* regulates stomatal development in tomato. **(A–C)** Stomatal index, stomatal density, and pavement cell density were determined in the abaxial epidermis of the mature third leaf from wild-type (MM) and *Slyda1* and *Slyda2* plants. The box plot diagram shows the median of the distribution as a center line, and the 25th and 75th percentiles are indicated by box limits. Whiskers show the minimum and maximum values. Data were obtained from 13 to 16 plants of each genotype, except for *Slyda1* lines #36 (*n* = 7) and #37 (*n* = 9). Pairwise phenotypic differences were analyzed by Mann-Whitney test, since data did not meet parametric test assumptions. Asterisks indicate statistical significance with respect to MM plants (^*^p ≤ 0.05, ^**^p ≤ 0.01, and ^***^p ≤ 0.001). **(D)** Representative differential interference contrast (DIC) images of the epidermis scored in panels **(A–C)** of the indicated genotypes. Stomata are false colored in blue. Bar = 50 μm. See also Supplementary Table S8.

To further confirm the functionality of the tomato *YDA* genes, we determined the impact of *SlYDA1* and *SlYDA2* loss of function on stomatal development, and we found that *Slyda2*, but not *Slyda1* lines, showed a higher stomatal index and occasional stomatal clusters (mainly paired stomata) in comparison to wild-type plants (Figure 6; Supplementary Table S8), indicating that *SlYDA2* is a negative regulator of stomatal formation and enforces correct stomatal patterning, as it has been described for *AtYDA* (Bergmann et al., 2004; Lukowitz et al., 2004). In the *Slyda2* lines, the higher stomatal index phenotype was associated with a significantly higher stomatal density, without changes on pavement cell density. In contrast, mutations in *SlYDA1* led to increased pavement cell density, which was translated into a higher stomatal density, but not in a higher stomatal index. These results suggest that *SlYDA1* and *SlYDA2* play distinct and specialized roles in stomatal development in tomato and that *Slyda2* mutants show similar developmental/disease resistance phenotypes to those described in *Arabidopsis yda* mutants (e.g., *yda11*; Sopeña-Torres et al., 2018).

## Discussion

Plants have a robust innate immune system that relies on several layers of pathogens perception that active defensive response to cope with pathogens colonization. PRRs, that are responsible of perceiving microbial MAMPs and host derived DAMPs, are widely distributed in the genomes of plant species. However, some PRRs are only present in the genomes of certain plant families, such as it occurs with the elongation factor receptor (EFR), an RLK involved in the perception of the bacterial EF-Tu MAMP that is exclusively present in Brassicaceae genomes (Lacombe et al., 2010). Due to the relevant roles of PRRs in pathogen perception and immune activation, the genetic transfer among plant species of PRRs, mainly RLKs, by transgenic overexpression has resulted in a successful biotechnological strategy to engineer broad-spectrum disease resistance in crops (Lacombe et al., 2010; Lu et al., 2015). Also, it has been shown that overexpression in some crops of PRRs of receptor like proteins (RLP) type, lacking the cytoplasmic kinase domain, from other species confers enhanced resistance to pathogens, such as it occurs in potato (*Solanum tuberosum*) expressing the *Arabidopsis* receptor-like protein 23 that recognizes the MAMP nlp20 from *Phytophthora infestans* (Albert et al., 2015). The success of these crop protection strategies relies on the highly conserved downstream components regulating MTI signaling pathways among plant species, which are activated upon MAMPs perception by heterologous overexpressed PRRs. In this context, MAPK cascades play a relevant role as they are convergent nodes of the signaling events that take place upon MAMP/DAMP recognition (Zhang et al., 2018). *Arabidopsis* genome has 80 annotated MAP3K, while tomato has 89 (Wu et al., 2014). MAPK signaling cascades are of paramount importance in activation of plant immunity, and accordingly they are major targets of pathogen effectors, such as the *P. syringae* HopAI1 and HopF2 or *P. infestans* PexRD2 effectors, to abolish the activation of plant defense responses (Zhang et al., 2007; King et al., 2014).

*YDA*, that encodes a MAP3K with very high similarity to members of the sterile 11/MAP/ERK kinase (STE11/MEKK) family, regulates several developmental processes (Bergmann et al., 2004) and a non-canonical immune pathway in *Arabidopsis* that upon activation can confer broad-spectrum disease resistance (Sopeña-Torres et al., 2018). In *Arabidopsis*, YDA functions downstream of the PRR complex formed by ER/ERLs/TMM/BAK1 to regulate both stomatal development and immune responses (Bergmann et al., 2004; Sopeña-Torres et al., 2018). The function of YDA in other plant species has not been determined, whereas it has been shown that ER RLK controls in crops, like tomato and rice, similar developmental processes to those regulated in *Arabidopsis*, but also some responses to stresses, like thermotolerance, making *ER* an attractive trait for breeding (Shen et al., 2015; Kwon et al., 2020). Moreover, the transcriptional factors (e.g., SPCH and MUTE) controlling ER-mediated responses (e.g., stomatal development) are conserved in crops like tomato, and the orthologs from *Arabidopsis* are functional in tomato (Ortega et al., 2019). Based on these previous data, it could be anticipated that YDA might regulate ER-mediated pathway in other plant species. This hypothesis has been corroborated here by overexpressing *AtCA-YDA* in tomato and by demonstrating the role of tomato orthologs of *AtYDA* (*SlYDA1* and *SlYDA2*) in regulating immune responses (i.e., *SlYDA1* and *SlYDA2*) and stomatal development (i.e., *SlYDA2*) in tomato plants.

To characterize the function of YDA in the regulation of crops immune responses, we have generated tomato lines that express the constitutive active MAP3K YDA from *Arabidopsis* (CA-YDA) that has been previously reported to confer broad-spectrum disease resistance to pathogens in *Arabidopsis* (Sopeña-Torres et al., 2018). Here, we demonstrate that heterologous expression of *AtCA-YDA* renders tomato plants more resistant to the bacterial pathogen *Pto* (Figure 3). These results strongly support the described function of YDA in plant immunity (Sopeña-Torres et al., 2018) that has been questioned by some previous results that described the antagonistic interaction of YDA on the immune pathway mediated by MAP3K3/5 (Sun et al., 2018). Remarkably, *AtCA-YDA* tomato lines (#3 and #7) showed a significant reduction in the stomatal index compared to wild-type plants (Figure 2), in accordance with the phenotype observed in *Arabidopsis CA-YDA* plants (Bergmann et al., 2004). All these results indicate that *AtCA-YDA* is functional in tomato plants and that all the downstream and upstream molecular components needed for the proper activation of the signaling pathway mediated by YDA, to control immune responses and stomatal development and patterning, are conserved between tomato and *Arabidopsis*.

Approaches to overexpress MAP3Ks in different plant species have been conducted previously to obtain plants with enhanced resistance to pathogens. However, many of these transgenic lines showed constitutive programed cell death activation, associated to the expression of SA-marker genes (e.g., *SlPR1*), and developed necrotic lesions on leaves that make unfeasible its possible biotechnological application as a strategy to obtain plants with enhanced resistance (Asai et al., 2002; del Pozo et al., 2004; Yamada et al., 2016). Notably, the tomato lines overexpressing *AtCA-YDA* protein do not show either developmental alterations or spontaneous necrotic lesions, and in accordance these lines do not have fitness or fruit yield penalties (Figure 1; Supplementary Figure S3). Therefore, overexpression of YDA in tomato might potentially be used as a strategy to increase disease resistance.

In *Arabidopsis*, deletion of certain amino acids from the N-terminal domain causes the constitutive activation of the MAP3K YDA (Lukowitz et al., 2004), and consequently a deep transcriptional reprograming and the constitutively upregulation of non-canonical defensive genes (Sopeña-Torres et al., 2018). Among the genes whose expression is upregulated by YDA activation in *Arabidopsis*, there are genes encoding antimicrobial peptides or R proteins (Sopeña-Torres et al., 2018). In tomato *AtCA-YDA* plants, at least 10.5% (34 genes) of the upregulated DEGs have a direct contribution to plant immune responses, and this set of DEGs includes some genes encoding antimicrobial peptides, such as thionins, lipid transfer proteins, chitinases, or cathepsin D inhibitors (García-Olmedo et al., 1998; Lisón et al., 2006). Besides, *AtCA-YDA* heterologous expression in tomato triggered the induction of three subtilases (e.g., *P69C*; Supplementary Table S2), which are extracellular proteases that have been described to exert a relevant role in responses to biotic stresses (Jordá et al., 1999; Schaller et al., 2018). In fact, overexpression of tomato *P69C* ortholog in *Arabidopsis* (*AtSBT3.3*) results in chromatin remodeling that concomitantly leads to an immune priming state (Ramírez et al., 2013). Interestingly, expression of some *R* genes was also upregulated on *AtCA-YDA* tomato plants (Figure 4; Supplementary Table S2). Though, it has been previously described that the constitutive overexpression of some *R* genes can result in constitutive upregulation of defense responses (Stokes and Richards, 2002), we did not find among the tomato *AtCA-YDA* DEGs defensive markers of canonical resistance pathways, like those regulated by the main defensive hormones (e.g., SA, ET, or JA). This is in line with our previous finding that the broad-spectrum resistance of *Arabidopsis AtCA-YDA* plants was not due to the activation of canonical immune pathways (Sopeña-Torres et al., 2018), further supporting that the ER-YDA signaling regulates a novel immune pathway. Notably, among the upregulated DEGs in tomato *AtCA-YDA* lines, there is a relevant number of genes that belongs to a functional category related with translation and protein folding (38.6% of genes), suggesting that the constitutive expression of *AtCA-YDA* might have an impact on cells reprograming for efficient protein translation. These data might support a function of YDA in the control of translation initiation in plants under biotic stress conditions, as it has been recently described for heat stress through YDA interaction with HSP90.1 protein (Samakovli et al., 2020). The transcriptional reprograming observed in tomato *AtCA-YDA* lines would explain the enhanced disease resistance observed in the transgenic tomato lines.

*Arabidopsis* has a unique *YODA* gene, while in rice and *Brachypodium* at least two *YDA* homologues have been identified (McKown and Bergmann, 2020). Tomato has at least three *YODA* like MAP3K close orthologs (Supplementary Figure S1) probably arisen by gene duplication and subsequent specialization. Using CRISPR strategy, we have been able to determine that *SlYDA1* is mainly involved in meristem development, as lines impaired in this member of the family showed aberrant phenotypes at early stages of development that are attenuated at later developmental stages (Supplementary Figure S7), while *SlYDA2* has functions, non-redundant with *SlYDA1*, in regulating stomatal development (Figure 6). Interestingly, mutations in *SlYDA1* and *SlYDA2* render plants highly susceptible to the bacterium *Pto* (Figure 5), indicating that both genes are relevant to activate a proper immune response that is independent of the SA, JA, and ET-mediated responses that are not defective in tomato CRISPR lines as revealed by the expression levels of marker genes of these canonical defensive pathways (Figure 5). However, the contribution of *SlYDA1* and *SlYDA2* to resistance seems to be different since *Slyda2* mutants are significantly more susceptible to *Pto* than *Slyda1* mutants. Future work will be required to unveiling their specific roles in tomato immunity, as well as the contribution of these two genes to tomato resistance to pathogens with other colonization styles. The stomatal development and immunity-associated phenotypes of *Slyda2* mutant lines resemble those of *Arabidopsis yda11* hypomorphic allele (Sopeña-Torres et al., 2018), suggesting that *SlYDA2*, like *AtYDA*, regulates stomatal formation and immunity. This would indicate that *SlYDA2* has in tomato a very similar function than *AtYDA* in *Arabidopsis*, which is in line with similarities of their N-terminal regulatory domains (Supplementary Figure S2A). We have previously suggested that regulation of these two processes (immune responses and stomatal development) was co-opted during evolution since appearance of stomata increased the potential entry points of pathogens in plants (Sopeña-Torres et al., 2018). The results obtained with *Slyda2* mutants and *AtCA-YDA* overexpressing lines are in accordance with this hypothesis.

*Arabidopsis* plants with different levels of expression of *AtYDA* show detrimental phenotypes, since *yda* null mutants (e.g., *yda 1*) are embryo lethal and do not complete life cycle, and plants overexpressing *AtCA-YDA* are viable just in heterozygosis (Bergmann et al., 2004; Sopeña-Torres et al., 2018). *Slyda1* mutant lines show some phenotype alterations at early stages of development (Supplementary Figure S7). Remarkably, tomato *Slyda2* and *AtCA-YDA* do not show alterations in fitness and fruit yield under our growth conditions, indicating that YDA might be a target trait for breeding crop protection, particularly in crops harboring several YDA orthologs in their genomes. Also, the identification of natural variants of *YDA* allele with increased activity of *SlYDA2* might have technological impact since we could obtain tomato plants with broad-spectrum disease resistance without fitness alterations and yield penalties.

## Data Availability Statement

RNA-seq read data have been submitted to the NCBI Sequence Read Archive (SRA) 283 under accession PRJNA635132.

## Author Contributions

AMo and LJ designed the study and wrote the manuscript with input from all authors. SS-T generated the *35S::AtCA-YDA* and *35S::AtYDA* constructs for tomato transformation. Plant Response Biotech (RP, YS, and MB) generated the Solanum transformed with *35S::AtCA-YDA* and *35S::AtYDA*. Semillas Fitó (TJ and AMa) generated the tomato CRISPR/Cas9 lines in collaboration with MN, who did the constructs. JT performed the phenotypic characterization of the transgenic lines and the validation of the RNA seq. JT and LJ did the pathogen infection experiments. LJ did the gene expression analysis after *Pto* inoculations. AM-F, AO, and MM performed the stomata counting assays. AM-B analyzed the RNAseq data. All authors contributed to the article and approved the submitted version.

### Conflict of Interest

AMo, LJ, and SS-T are inventors on the patent (US14/652,285) claiming the use of YDA for improving crop resistance. AMo is member of the scientific advisory board of Plant Response Biotech S.L. Authors RP, YS, and MB were employed by the company Plant Response Biotech, and AMa and TJ were employed by the company Semillas Fitó.

The remaining authors declare that the research was conducted in the absence of any commercial or financial relationships that could be construed as a potential conflict of interest.

## Footnotes

^1^https://ptf.ucdavis.edu/services

^2^http://bioinfogp.cnb.csic.es/tools/breakingcas/

^3^imed.med.ucm.es
