# Supplementary material for: YODA Kinase Controls a Novel Immune Pathway of Tomato Conferring Enhanced Disease Resistance to the Bacterium Pseudomonas syringae
Source: Front Plant Sci. 2020 Oct 14;11:584471. doi: 10.3389/fpls.2020.584471 (PMC7591502; doi:10.3389/fpls.2020.584471)
Supplement: Supplementary file 1 [file Data_Sheet_1.pdf]

Supplementary Figure 1

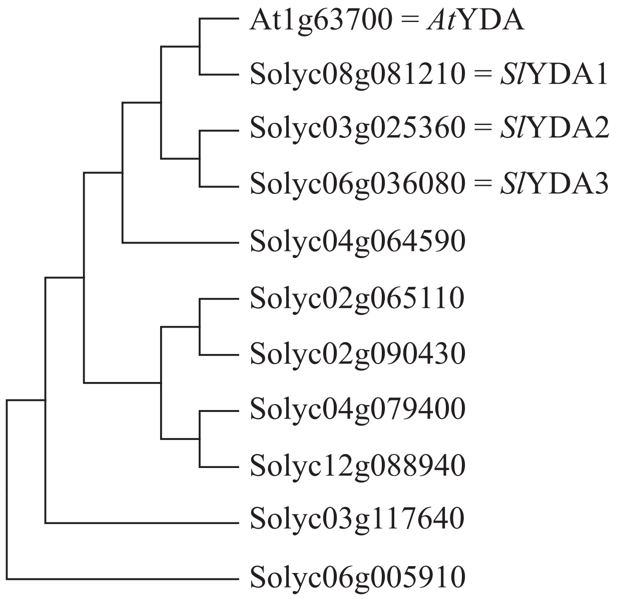

Supplementary Figure 1. Phylogenetic analysis of *At*YDA and tomato *Sl*YDA-like MAPKKKs. Phylogenetic analysis using the Neighbor-Joining method based on the full-length protein sequence of *Arabidopsis thaliana At*YDA1 (At1g63700) and putative orthologs from *Solanum lycopersicum Sl*YDA1 (Solyc08g081210), *Sl*YDA2 (Solyc03g025360), *Sl*YDA3 (Solyc06g036080). The tree also includes 6 members of the MAP3K family from *Solanum lycopersicum* (Solyc04g064590, Solyc20g065110, Solyc02g090430, Solyc04g079400, Solyc12g088940 and Solyc03g117640). Solyc06g005910 (coding for a tubulin β-chain) is used as an outlier.

Supplementary Figure 2

(A)

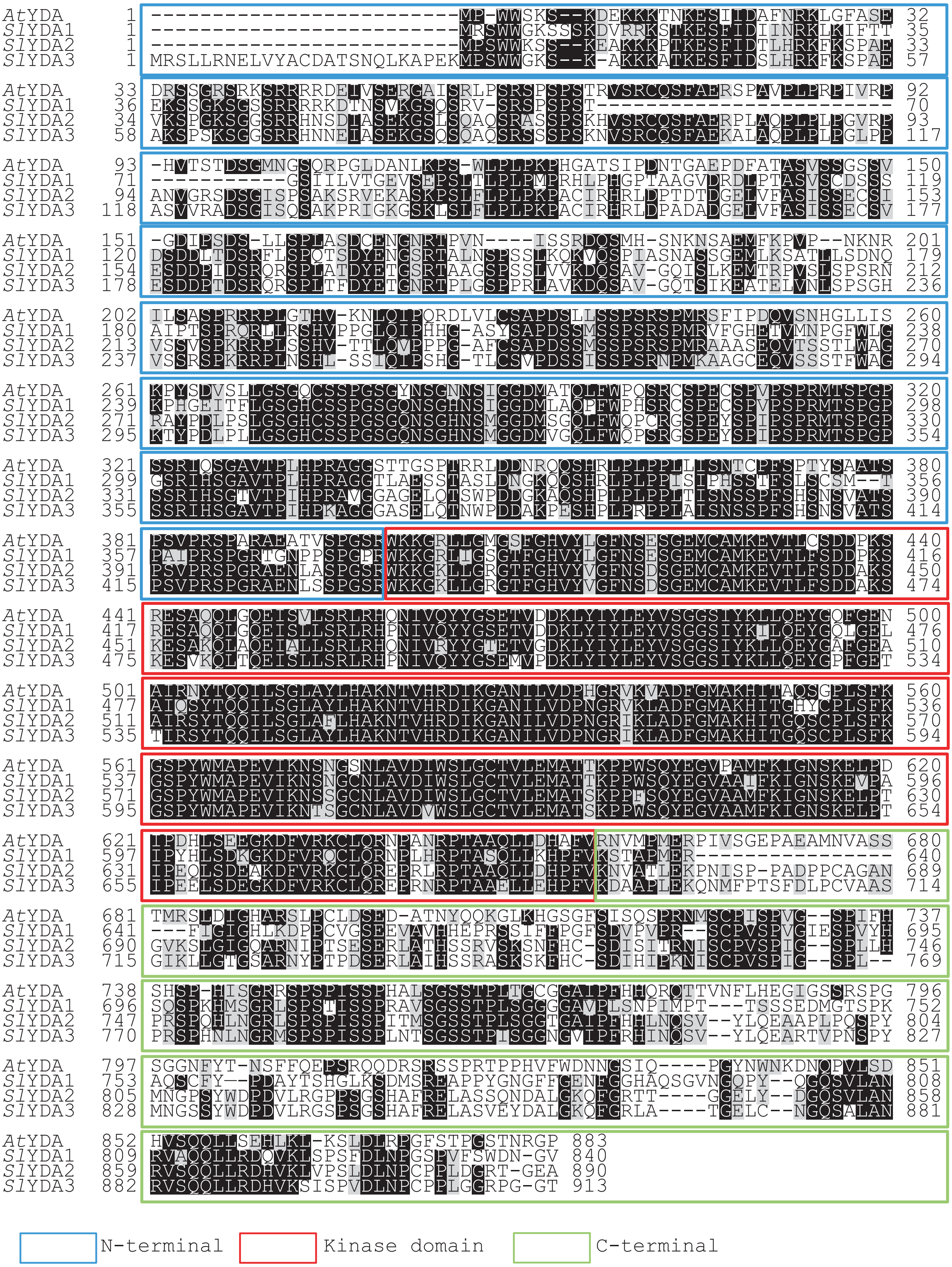

(B)

IDENTITY				
**At1g63700**	100%			
***Sl*YDA1**	60.71%	100%		
***Sl*YDA2**	59.22%	60.83%	100%	
***Sl*YDA3**	57.19%	59.64%	81.79%	100%
	**At1g63700**	***Sl*YDA1**	***Sl*YDA2**	***Sl*YDA3**

(C)

SIMILARITY				
**At1g63700**	100%			
***Sl*YDA1**	67.5%	100%		
***Sl*YDA2**	67.15%	66.9%	100%	
***Sl*YDA3**	65.23%	65.95%	85.5%	100%
	**At1g63700**	***Sl*YDA1**	***Sl*YDA2**	***Sl*YDA3**

Supplementary Figure 2. Protein sequence alignment of *At*YDA and putative orthologs from *Solanum lycopersicum Sl*YDA1 (Solyc08g081210), *Sl*YDA2 (Solyc03g025360) and *Sl*YDA3 (Solyc06g036080). **(A)** Full length protein multiple sequence alignment by MUSCLE using MEGA. **(B)** Identity and **(C)** similarity results between the protein sequences using Sequence Identity And Similarity (SIAS) tool and BLOSUM62 matrix (imed.med.ucm.es).

Supplementary Figure 3

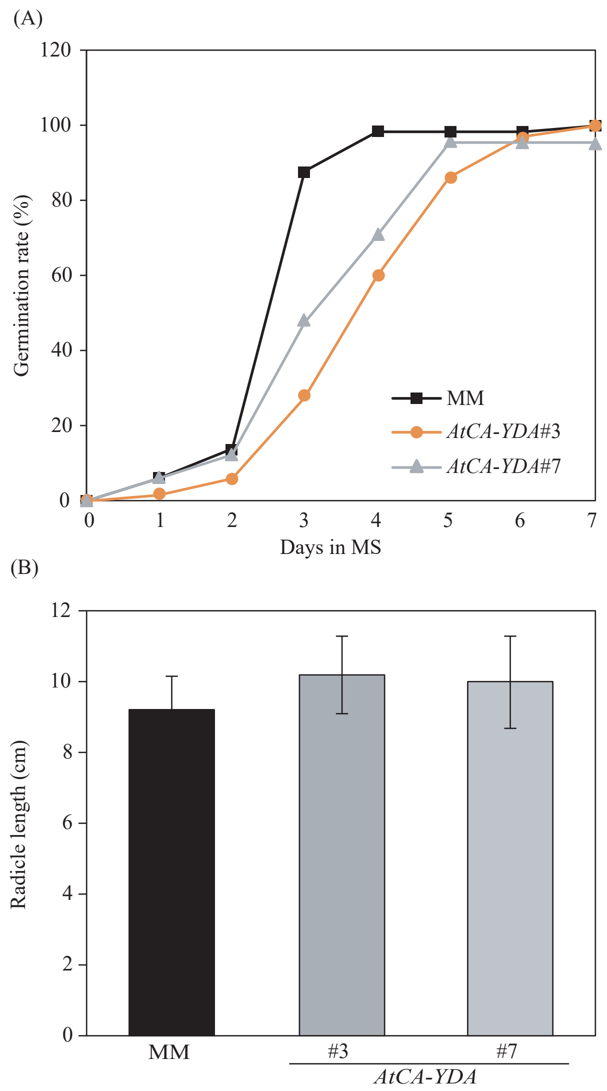

Supplementary Figure 3. Transgenic overexpression of *AtCA-YDA* in tomato does not have a significant effect on root development. **(A)** Germination rate of the different tomato lines grown in MS media in dark conditions. **(B)** Measurements of radicle lengths of the indicated lines 7 days after germination in MS media. Data shown are average (n=8) ± SE. Asterisks indicate statistical differences with respect to MM plants (Student´s *t*-test; * p-value < 0.05). These assays were performed at least three times with similar results.

Supplementary Figure 4

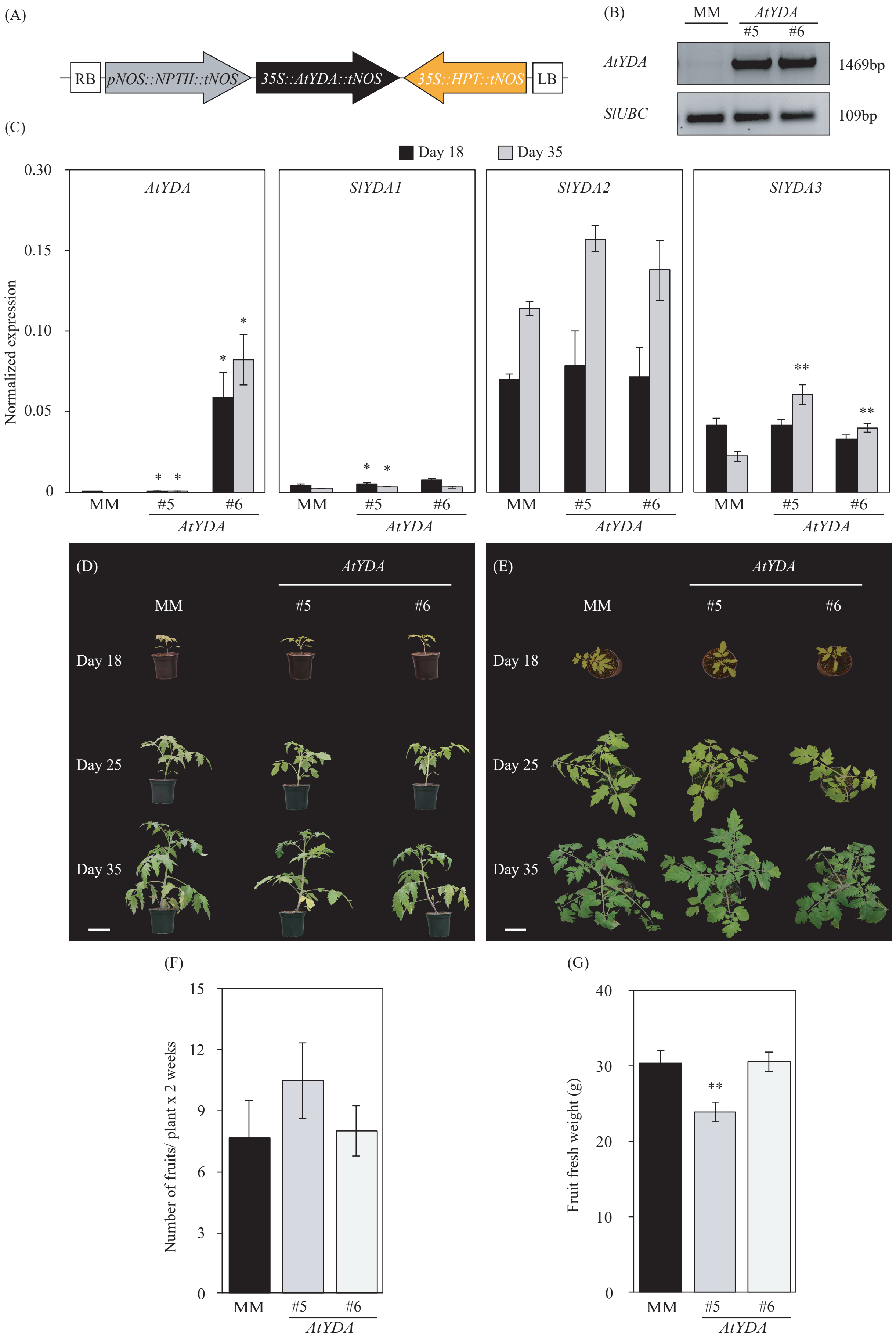

Supplementary Figure 4. Transgenic over expression of *AtYDA* in tomato does not affect tomato development. **(A)** Scheme of construction used to transform tomato plants with *AtYDA* using pGWB2 vector, driven by the *CaMV 35S* promoter (35S) and nopaline synthase terminator (tNOS). RB, right border sequence of T-DNA; LB, left border sequence of T-DNA; *NPTII*, aminoglycoside phosphotransferase from Tn5 driven by nopaline synthase promoter (*pNOS*) and nopaline synthase terminator (*tNOS*); *HPT*, hypoxanthine-guanine phosphoribolsyltransferase gene driven by *35S* promoter and *tNOS* terminator. **(B)** Detection by PCR of the transgene in the indicated Moneymaker (MM) and *AtYDA* #5 and *AtYDA* #6 transgenic lines. PCR amplified *SlUBC* was used as equal DNA loading control. Of note *AtYDA* #5 does not harbour the transgene and it is included as control. **(C)** Expression of *AtYDA, SlYDA1* (*Solyc08g081210*), *SlYDA2* (*Solyc03g025360*) and *SlYDA3* (*Solyc06g036080*) in *S. lycopersicum* MM and transgenic *AtYDA* plants (lines #5 and #6) at 18 and 35 days after sowing. Shown are the averages of three independent experiments (n=3) ± SE of normalized expression. Asterisks indicate statistical significance with respect to MM plants (Student´s *t*-test; * p-value < 0.05; ** p-value < 0.01). **(D-E)** Morphology of the indicated genotypes at 18, 25 and 35 days after sowing. Bar = 10 cm. **(F)** Number of fruits collected per plant from the indicated lines (n=12). **(G)** Fresh weight (in g) of the tomato fruits produced by the transgenic lines (#5 n=92; #6 n=72) and the Moneymaker (n=58) plants (MM). Student´s *t*-test; * p-value < 0.05; ** p-value < 0.01.

Supplementary Figure 5

(A)

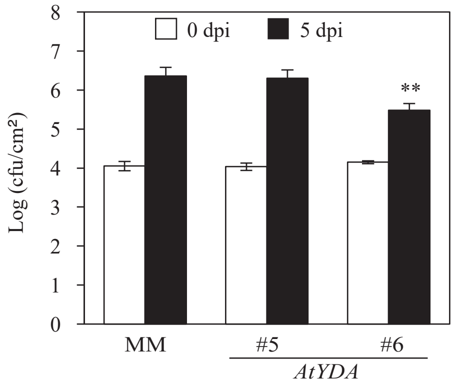

(B)

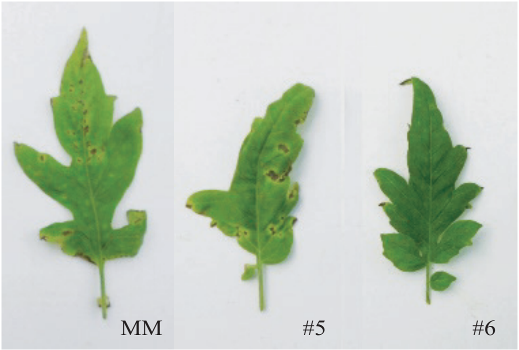

Supplementary Figure 5. Heterologous expression of *AtYDA* into tomato plants confers enhanced resistance to *Pseudomonas syringae* pv. tomato DC3000. **(A)** Quantification of bacterial growth at 0, and 5 days post-inoculation (dpi). Values represent the mean (n=16) ± SE of three independent experiments. Asterisks indicate values statistically significant with respect to MM inoculated plants (Student´s *t*-test; * p-value < 0.05; ** p-value < 0.01). **(B)** Macroscopic disease symptoms on tomato leaves inoculated with the bacterium at 8 dpi. This experiment has been performed at least three times with similar results.

Supplementary Figure 6

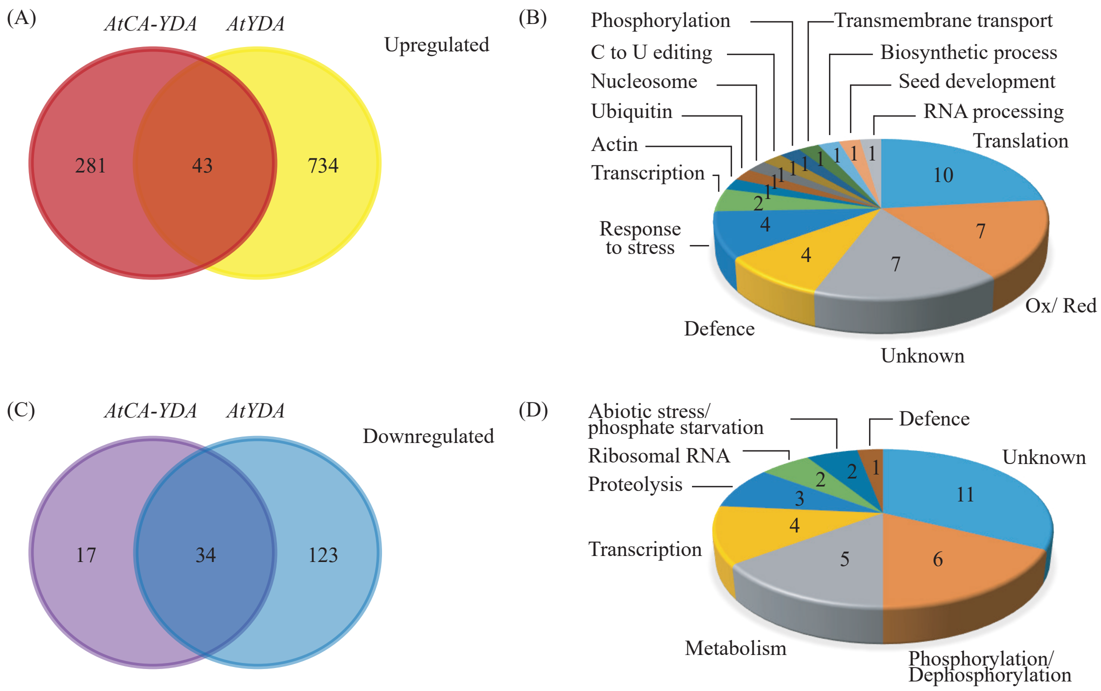

Supplementary Figure 6. Functional classification of differentially miss-regulated genes in *AtYDA* and *AtCA-YDA* overexpressing lines. **(A)** Venn diagram showing overlapping of genes commonly upregulated in *AtCA-YDA* lines (#3 and #7) and in *AtYDA* plants compared to Moneymaker wild-type plants. Differential expression was defined as at least 2-fold change when compared with the non-transformed controls (MM). A minimal coverage value of 20 for each gene in RNAseq data was considered for selecting the genes. **(B)** Functional category classification of the 43 core genes up-regulated in *AtCA-YDA* and *AtYDA* tomato lines. BINGO pipeline was used to assign the categories. See Table S6 for further information on the 43 core genes up regulated. **(C)** Venn diagram showing the overlapping of genes downregulated in *AtCA-YDA* lines (#3 and #7) and *AtYDA* plants compared to Moneymaker. **(D)** Functional category classification of the 34 down-regulated core genes found in the *AtCA-YDA* and *AtYDA* tomato lines using BINGO pipeline. Table S7 shows the 34 core genes downregulated.

Supplementary Figure 7

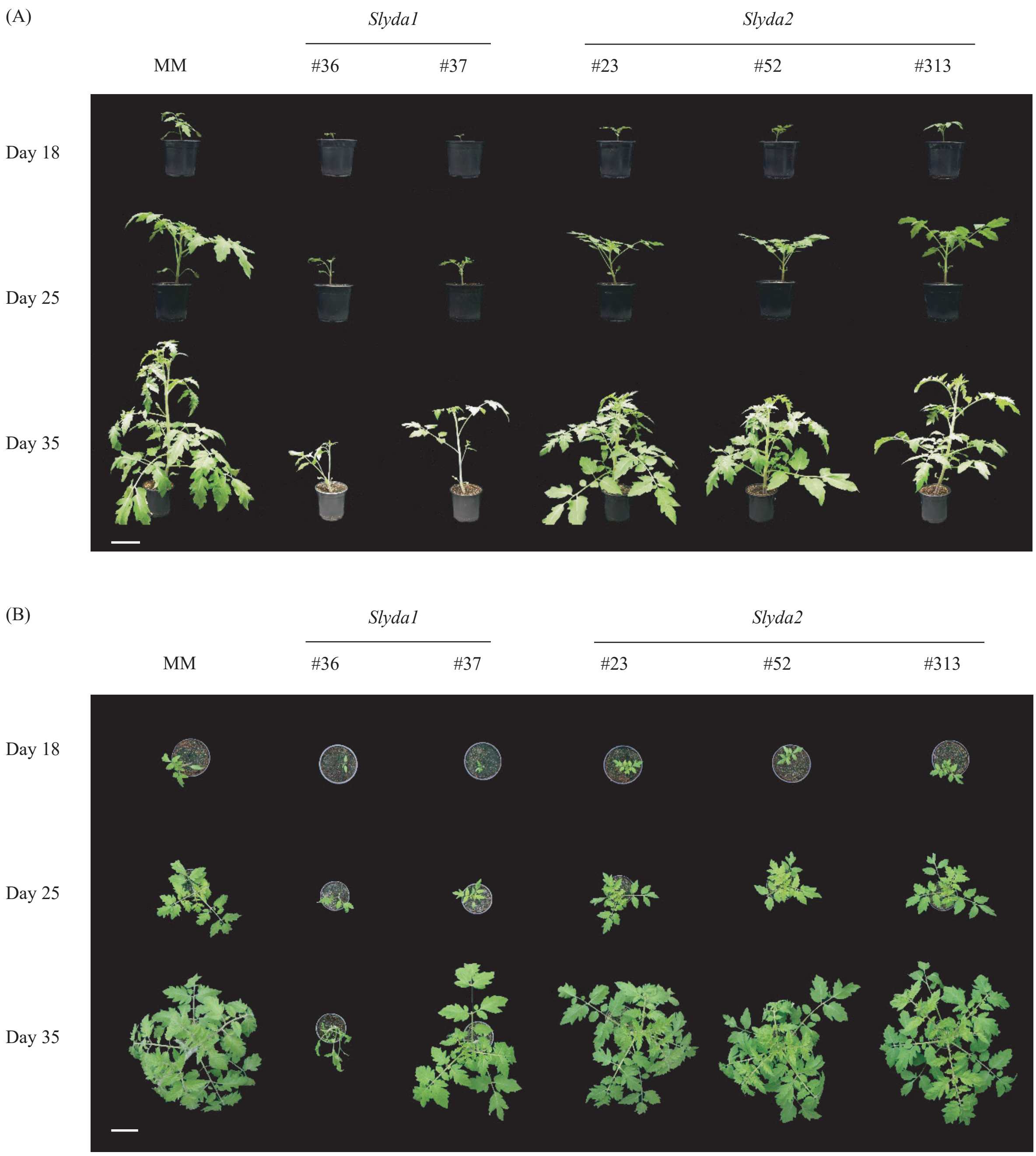

Supplementary Figure 7. Phenotypic characterization of leaf and stem development of tomato *Slyda1* and *Slyda2* mutants. **(A)** Frontal view of MM and CRISPR-edited tomato lines at 18, 25 and 35 days after sowing. Bar = 10 cm. **(B)** Aerial view of the indicated genotypes at 18, 25 and 35 days after sowing. Bar = 10 cm.
